# A new species of ***Newportia*** Gervais, 1847 from Puerto Rico, with a revised key to the species of the genus (Chilopoda, Scolopendromorpha, Scolopocryptopidae)

**DOI:** 10.3897/zookeys.276.4876

**Published:** 2013-03-08

**Authors:** Arkady A. Schileyko

**Affiliations:** 1 Zoological Museum of Moscow State Lomonosov University, Bolshaja Nikitskaja Str.6, 103009, Moscow, Russia

**Keywords:** *Newportia*, Newportiinae, new species, identification key, external characters

## Abstract

A new species of the centipede genus *Newportia*, *Newportia stoevi* **sp. n.**, is described from Rio Encantado Cave, Puerto Rico. It differs from all congeners by having sternites distinctly margined laterally and ultimate legs bearing 4 spinous processes on both prefemur and femur, and 2 on tibia. The value of some terms used in the taxonomy of the genus have been analyzed and an amended identification key to the species of *Newportia* is provided.

## Introduction

The genus *Newportia* Gervais, 1847 is still poorly known. It is especially so with regards to the Puerto Rican fauna where only two species have hitherto been registered. Silvestri (1908) reported *Newportia ernsti* Pocock, 1891 from Coamo Springs, while Chagas and Shelley (2003) recorded *Newportia heteropoda* Chamberlin, 1918 from two localities – 8.4 mi (13.4 km) SW Luquillo, trail to Minas Falls off hwy. 191, Luquillo Division, Caribbean National Forest and from 4 mi (6.4 km) N Villalba, Dona Juaña Recreation Area. To these should be added Chamberlin’s (1950) uncertain record of *Newportia* sp. from Maricao Insular Forest, based on a specimen with missing ultimate legs.

Herewith, I describe a new species of *Newportia* recently collected in Puerto Rico by Dr Petar Beron from the National Museum of Natural History, Sofia (NMNHS). The specimen was assigned to *Newportia* and tentatively identified as a new species by Dr Pavel Stoev, curator of Myriapoda at the NMNHS who committed it for further study to me. This specimen differs from all congeners, in the first place, by important traits of the ultimate legs (which are normally developed and have no traces of regeneration) and unusually developed lateral margination of sternites.

The identification key to the species of *Newportia* (Schileyko and Minelli 1998) has been updated to accommodate this and other new species described recently (e.g., *Newportia troglobia* Chagas & Shelley, 2003), as well as to reflect other nomenclature novelties proposed in the genus. Some general notes on the external anatomy of *Newportia* have been made, too. The terminology follows Bonato et al. (2010).

## Systematic part

### SCOLOPENDROMORPHA Pocock, 1895

Scolopocryptopidae Pocock, 1896

Newportiinae Pocock, 1896

*Newportia* Gervais, 1847

#### Newportia stoevi sp. n.

urn:lsid:zoobank.org:act:AE5E2F31-F3F2-45EB-9AD6-24EDA31D1D66

http://species-id.net/wiki/Newportia_stoevi

Figs 1 [Fig F2] 10

##### Holotype:

Puerto Rico, Florida Co., Rio Encantado Cave, 1 (sub?)adult, 29.07.2009, leg. P. Beron (NMNHS).

##### Locus typicus.

Puerto Rico, Florida Co., Rio Encantado Cave.

##### Derivatio nominis:

named after my friend and colleague Dr Pavel Stoev who drew my attention to this new species.

##### Diagnosis.

Tergite 1 with rounded anterior transverse suture and incomplete paramedian sutures. Sternites distinctly margined laterally. Ultimate legs: prefemur with 4, femurwith 3 small spinous processes medially and 1 ventrally; tibia with 2 small spinous processes medially. Tarsus 1 large and clavate (bulbous), clearly differing from the much thinner tarsus 2; the latter consisting of 19–20 articles.

##### Description.

**Figures 1–4.**
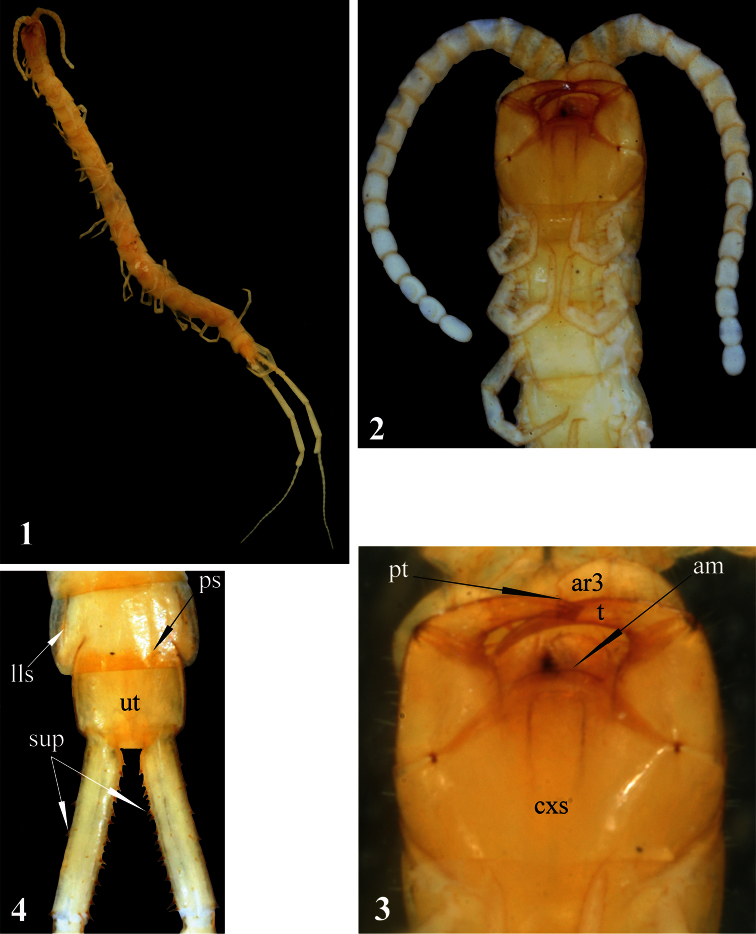
*Newportia stoevi*,sp. n. **1** Habitus **2** Head and anterior segments, ventral view **3** Forcipular segment, ventral view **4** Tergites 22 and 23 and prefemora of ultimate legs, dorsal view; (**pt**) – pretarsus of second maxilla, (**ar3**) – article 3 of telopodite of second maxilla, (**cxs**) – forcipular coxosternite, (**am**) – anterior margin of coxosternite, (**t**) – tarsungulum, (**ps**) – paramedian sutures, (**lls**) – lateral longitudinal sutures, (**ut**) – tergite of ultimate leg-bearing segment, (**sup**) – spurs of ultimate prefemur.

Length of body *ca* 17 mm, length of ultimate legs about 9 mm. Color (in ethanol): entire animal uniformly light-yellow with cephalic plate and forcipular segment slightly darker (Fig. 1). Body sparsely pilose; sternites and legs less setose than tergites.

Antennae composed of 17 articles (Fig. 2), reaching rear edge of tergite 5 when folded backwards; 2.5 basal antennal articles covered by a few long setae, subsequent articles densely pilose. Basal antennal articles somewhatflattened dorso-ventrally.

Head: cephalic plate visibly longer than wide, with rounded corners and very short paramedian sutures at posterior margin.

Second maxillae: as in all other *Newportia* species but dorsal spur on article 2 of the telopodite not recognisable. Pretarsus without spurs, with well-developed dorsal brush. The angle between the longitudinal axes of pretarsus and article 3 of telopodite slightly more than 100° (Fig. 3), which is quite unusual condition in Scolopendromorpha.

Forcipular segment: coxosternite without any visible sutures (including thechitin-lines). Anterior margin of coxosternite evidently convex (Fig. 3), divided by a median diastema into two low additionally sclerotised lobes; each lobe bearing a longseta. Trochanteroprefemoral process absent. Tarsungula normal.

Tergites: anterior margin of tergite 1 covered by the cephalic plate; tergite 1with a rounded anterior transverse suture and paramedian sutures stretching from the transverse suture to the posterior tergal margin. Tergite 3 with a very characteristic thin oblique sutures bordering the anterior corners of tergite. Tergites 2-22 with complete paramedian sutures, tergites 3-21(22)withlateral longitudinal sutures (Fig. 4). Tergite 23 lacking sutures, its posterior margin convex. Tergite margination virtually absent, only tergite 23 distinctly margined laterally.Tergite 23 much wider rather than long and nearly rectangular in shape; its lateral sides slightly rounded (Fig. 4). All tergites without medial keel; pretergites also missing.

**Figures 5–8. F2:**
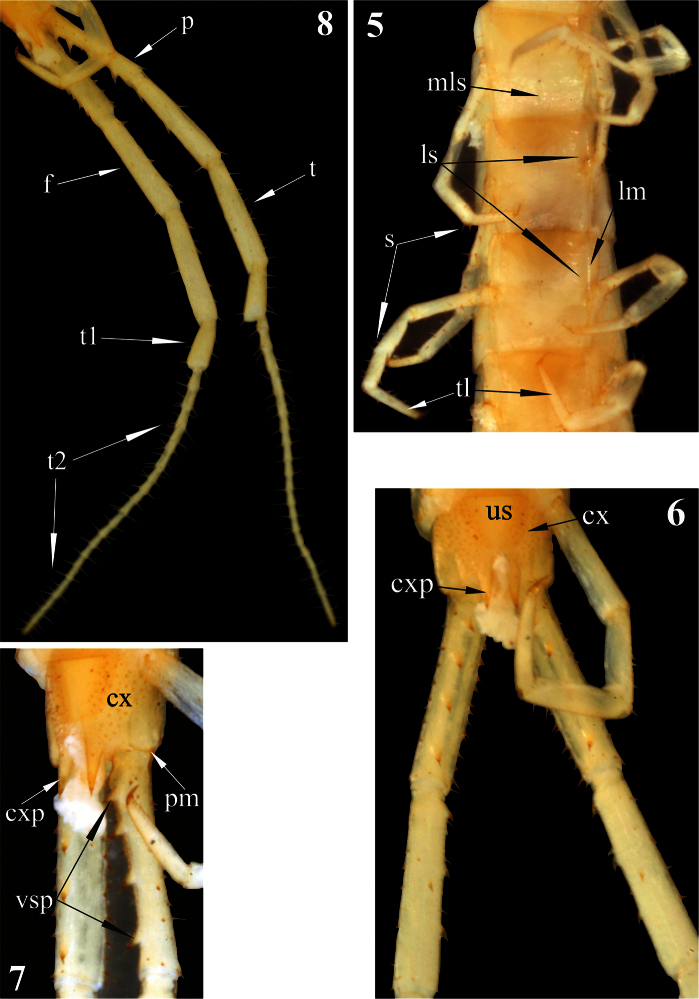
*Newportia stoevi*,sp. n. **5** Segments and midbody legs, ventral view **6** Posterior body end, ventral view **7** Left side of ultimate leg-bearing segment and prefemora of ultimate legs, ventro-lateral view **8** Ultimate legs, ventro-lateral view; (**mls**) – median longitudinal sulcus, **(ls) –** lateral sutures, (**lm**) – lateral margination, (**s**) – setae, (**tl**) – monoarticulated tarsus of locomotory leg, (**us**) – sternite of ultimate leg-bearing segment, (**cx**) – coxopleuron, (**cxp**) – coxopleural process, (**pm**) – posterior margin of pleuron of ultimate leg-bearing segment, (**vsp**) – ventral spinous processes of ultimate prefemur, (**p**) – prefemur, (**f**) – femur, (**t**) – tibia, (**t1**) – tarsus 1, (**t2**) – tarsus 2.

Sternites: trapeziform, 2-22 with incomplete (equally shortened from both sides) but with a well expressed median longitudinal sulcus. Sternites 2-21 with definite and complete lateral margination (Fig. 5) through lateral longitudinal sutures (see Remark 2); endosternites absent. Sternite 23 trapeziform, with a few very short (spur-like) setae on lateral sides (Fig. 6), with a straight posterior margin.

Legs: prefemur, femur and tibia with a few large setae (Fig. 5); tarsi with more numerous setae of various length and size. Tibia of legs 1–20 with a lateral spur; both, ventral tibial spur and tarsal spur absent. Tarsi of legs 1–21 (Fig. 5) without distinct division between tarsus 1 and 2; pretarsi long, thin and sharply pointed.Pretarsi of legs 1-22 with two thin and long (as long as 1/2 of pretarsus)accessory spines.

Coxopleuron (Figs 6, 7):nearly completely pierced with coxal pores of various size *–* only coxopleural process and a narrow area bordering posterior margin of coxopleuron remaining poreless. Coxopleural process (Figs 6, 7)as long as ultimate sternite, conical, without additional spines. Coxopleural surface without setae.Posterior margin of pleuron of ultimate leg-bearing segment forming avery obtuse angle.

**Figures 9–12.**
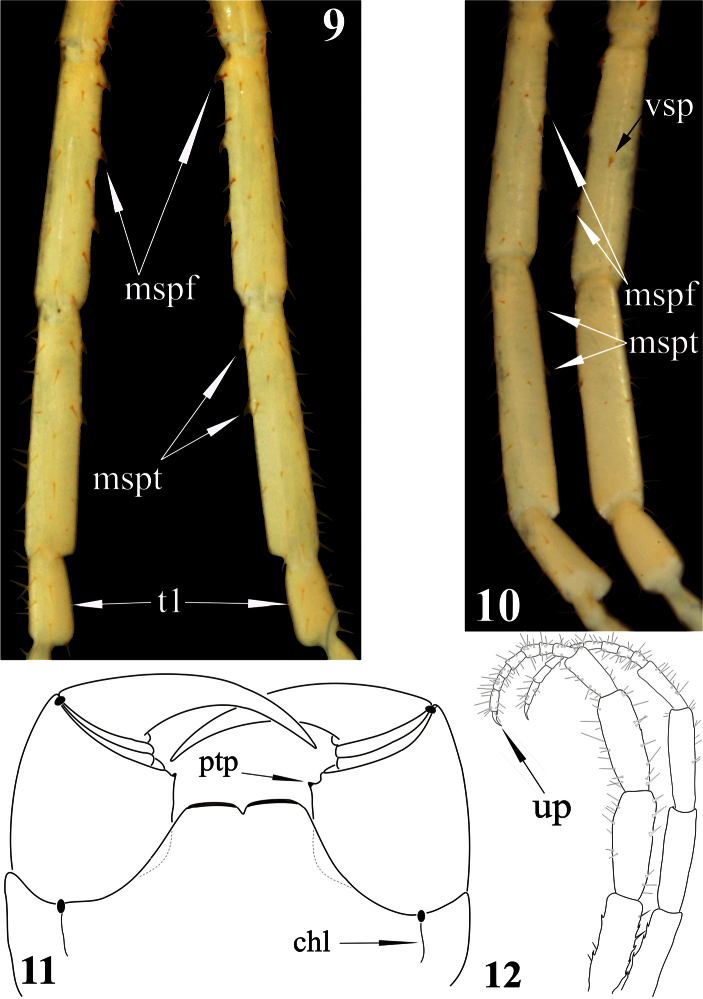
*Newportia stoevi*,sp. n. **9** Femora, tibiae and tarsi 1 of ultimate legs, dorsal view **10** Femora, tibiae and tarsi 1 of ultimate legs, ventral view; *Newportia divergens* Chamberlin, 1922 **11** Forcipular segment, ventral view (after Schileyko and Minelli 1998); *Newportia unguifer* Chamberlin, 1921 **12** Ultimate legs, dorso-lateral view (after Schileyko and Minelli 1998); (**mspf**) – medial spinous processes of ultimate femur, (**mspt**) – medial spinous processes of ultimate tibia, (**vsp**) – ventral spinous process of ultimate femur, (**t1**) – tarsus 1, (**up**) – ultimate pretarsus, (**chl**) – chitin-lines, (**ptp**) – process of trochanteroprefemur.

Ultimate legs (Fig. 8): slender, *ca* 9 mm long, width of prefemur *ca* 0.5 mm.Prefemur triangular in cross-section, with a standard row of 4 ventral spinous processes (Fig. 7), some spurs (strong, spine-like setae of various length) dorso-laterally and more numerous similar spurs dorso-medially (Fig. 4). All four prefemoral ventral spinous processes are of the same size, apically curved and ending in a pointed harpoon-like tip, which is accompanied by a long seta. Femurcylindrical,with 3 small spinous processes medially (Fig. 9) and 1 ventrally in the middle of femur (Fig. 10). Tibia cylindrical, with 2 small spinous processes medially: one close to its base and another at mid length (Figs 9, 10). Both femoral and tibial spinous processes are accompanied by a single long ventral seta. Tibia practically as long as prefemur or femur. Tarsus well divided into tarsus 1 and tarsus 2 (Fig. 8), former as long as 1/2 of tibia. Tarsus 1 (Figs 8–10) is enlarged and clavate (bulbous); tarsus 2 thin, consisting of 19 (or 20) articles (Fig. 8). In a few places annulation of tarsus 2 is somewhat vague; for example, the very long ultimate article seems to consist of two articles, which are not well divided. Ultimate legs without pretarsus.

##### Range.

The species is hitherto known only from its type locality.

##### Habitat and associated fauna.

Being -250 m deep and 16 910 m long Rio Encantado is the deepest and the longest cave system in Puerto Rico. This system lies in the Tertiary limestone area which stretches along the northern coast of the island (Peck 1974). *Newportia stoevi* has been collected deep inside the cave, in the aphotoc zone and although apparent troglomorphic traits are lacking it may well represent a troglobite, as its congener from Sistema de Purificacion, Mexico, *Newportia troglobia* (Chagas and Shelley 2003). In the cave it co-occurs with amblypigs, spiders, beetles (Dr. P. Beron, pers. comm.).

## Discussion

1 This species is morphologically close to *Newportia heteropoda* Chamberlin, 1918 from which it can be readily distinguished by the following traits of the ultimate pair of legs: number of articles of tarsus 2 (19 vs. 9 in *Newportia heteropoda*); presence of 4 (vs. 3 in *Newportia heteropoda*) spinous processes on femur; presence of 2 (vs. 0 in *Newportia heteropoda*) such processes on tibia.

2 Schileyko (2009) wrote that in the family Scolopocryptopidae the lateral sternal sutures are known in *Newportia*, *Tidops* Chamberlin, 1915, *Kartops* Archey, 1923, *Kethops* Chamberlin, 1912 and *Ectonocryptoides* Shelley & Mercurio, 2005. It should be noted, that Chagas (2011) considered *Kartops* as a junior synonym of *Tidops*. The lateral sternal sutures may be developed in various degrees (from complete to quite short), but only *Kethops* (see fig. 144 in Shelley 2002) and *Ectonocryptoides sandrops* Schileyko, 2009 have sterna with elevated lateral margins. However, in *Newportia stoevi* the lateral longitudinal sutures border the complete lateral margination, which seems to be considerably elevated over the surface of sternite.

3 Some groups of scolopendromorphs (the majority of Scolopendrinae, Otostigminae, Scolopocryptopinae and Plutoniumidae) have a well-developed, strongly sclerotized disto-medial projection of the forcipular trochanteroprefemur. Formerly, I used the term “forcipular median tooth” for it, but Bonato et al. (2010) proposed the term “process of trochanteroprefemur”. The Newportiinae either entirely lack this process, or have it only as a small denticle (Fig. 11), similar to some geophilomorphs, for which Bonato et al. (2010) proposed the term ‘distal denticles of trochanteroprefemur’.

4 As for the vague annulation of some articles of ultimate tarsus 2 in *Newportia stoevi*, I should mention that there are a few other species of *Newportia* in which this trait is observed, for example *Newportia albana* Chamberlin, 1957 and *Newportia diagramma* Chamberlin, 1921 (see REMARKS to *Newportia albana* and Figure 5c of *Newportia diagramma* in Schileyko and Minelli 1998).

5 In some species of *Newportia* legs have one tarsal spur and two (lateral and ventral) tibial spurs, other species have one (lateral) tibial spur only (as *Newportia stoevi*) and in *Newportia phoretha* Chamberlin, 1950 spurs are entirely lacking (see p. 290 in Schileyko and Minelli 1998). In some species of *Newportia* (for example in *Newportia longitarsis stechowi* Verhoeff, 1938) lateral tibial spur is situated on an outgrowth of disto-lateral side of the tibia (see Fig. 2a in Schileyko and Minelli 1998). It is also worth mentioning that tibial spurs do not break off easily in *Newportia* as these spurs would do, for example, in *Otostigmus*. Absence of tibial spurs is another character that separates Ectonocryptopinae from Newportiinae.

### Identification key to the species of *Newportia*

One of the main problems for identification of scolopendromorph centipedes is the high number of new species, described in the last decades that are still remaining outside the contemporary identification keys. I suggest that every description of new species in large genera (like *Newportia*) to be accompanied by the respective update of the available identification key. In cases where the genus includes just a few species, the identification key should be completely re-written.

The most recent key to the species of *Newportia* was provided by Schileyko and Minelli (1998). Since then several new species have been described by González-Sponga (1997, 2000) and Chagas and Shelley (2003) from Venezuela and Mexico, respectively. The latter authors have also revived *Newportia azteca* Humbert & Saussure, 1869, although in the same paper they also stated (pp. 13-14): “We … do not think that any conclusion [about the validity of *azteca*] can be reached”. In 1998 Schileyko and Minelli wrote (p. 291): “Another nominal taxon very similar if not identical to *Newportia oriena* and *Newportia spinipes* seems to be *Newportia azteca* Humbert & Saussure, 1869: 158 [cf. Attems, 1930: 275] whose true identity, however, remains to us as doubtful as it was to Attems [1930]”. However, Chagas and Shelley (2003) were absolutely correct when writing (p. 13) that *Newportia azteca* is the third oldest name in *Newportia* (after *Newportia longitarsis* and *Newportia mexicana*) and in case of synonymy would have priority by 27 years over *Newportia spinipes*. Since there is no available characters at the moment to separate these two species I put them together in the following identification key. Both, *Newportia stoevi* sp. n. and *Newportia troglobia*, are included in the key provided below. With regards to the seventeen new species of *Newportia* described from Venezuela by González-Sponga (1997, 2000), they will be analyzed in a paper dedicated to the scolopendromorph fauna of Venezuela that is currently in progress.

**Table:** 

1	Tarsus 2 of ultimate legs clearly divided into distinct articles	2
–	Tarsus 2 of ultimate legs undivided	31
2	Ultimate leg with a well-developed (claw-shaped) pretarsus which is as long as, or longer than half of the ultimate article of tarsus 2 (Fig. 12)	*Newportia unguifer*
–	Ultimate leg without a well-developed pretarsus	3
3	Tergite 1 without an anterior transverse suture	*Newportia sargenti*
–	Tergite 1 with an anterior transverse suture	*4*
4	Tergite 1 with a rounded anterior transverse suture and, generally, with paramedian sutures which do not form a “W” just behind the anterior transverse suture; in a few species these sutures are absent or extremely short (Fig. 13)	5
–	Tergite 1 with an anterior transverse suture in the form of a very obtuse angle and with paramedian sutures forked anteriorly, thus forming a “W” just behind the anterior transverse suture (Fig. 14)	21
5	Some pairs of legs, usually 2(4)-(19)20, with tibial spurs	6
–	Tibial spurs missing on all legs	*Newportia phoretha*
6	Femur of ultimate legs without spinous processes	7
–	Femur of ultimate legs with spinous processes	10
7	Tergite 1 with rudimentary paramedian sutures (Fig. 13) or sutures completely lacking	*Newportia pusilla*
–	Paramedian sutures of tergite 1 half-complete or complete, sometimes shortly interrupted in the middle	8
8	Coxopleural process extremely short; tergite 1 with poorly developed paramedian sutures (Fig. 15) which cross the anterior transverse suture	*Newportia diagramma*
–	Coxopleural process normal (Fig. 7); tergite 1 with well-developed paramedian sutures stretching between anterior transverse suture and posterior tergal margin	9
9	Tarsus 2 of ultimate legs composed of 19–25 articles	*Newportia aureana*
–	Tarsus 2 of ultimate legs composed of 6–7 articles	*Newportia longitarsis tropicalis*
10	Tibiae 2-20 with lateral and ventral spurs	11
–	Tibiae 2-20 with a lateral spur only	12
11	Femur of ultimate legs with 1(-2) ventral spinous process(es)	*Newportia cubana*
–	Femur of ultimate legs with 2-3 medial spinous processes	*Newportia longitarsis virginensis*
12	Tarsus 2 of ultimate legs composed of 4 articles; tarsus 1 almost as long as the tibia	*Newportia dentata*
–	Tarsus 2 of ultimate legs composed of 7-26 articles; tarsus 1 quite shorter than the tibia	13
13	Tarsus 2 of ultimate legs composed of 26 articles; cephalic plate without paramedian sutures	*Newportia leptotarsis*
–	Tarsus 2 of ultimate legs composed of 7–20 articles; cephalic plate often with incomplete paramedian sutures	14
14	Anterior ends of the half-complete paramedian sutures of tergite 1 very shortly bifurcate behind the anterior transverse suture (Fig. 16)	*Newportia oligopla*
–	Paramedian sutures of tergite 1 from absent to complete, never bifurcated anteriorly	15
15	Tarsus of ultimate legs uniformly divided, without distinction into tarsus 1 and tarsus 2	*Newportia adisi*
–	Tarsus of ultimate legs distinctly divided into tarsus 1 and tarsus 2	16
16	Femur of ultimate legs with 3-4 spinous processes	17
–	Femur of ultimate legs with 1-2 spinous processes	18
17	Femur of ultimate legs with 3 spinous processes, tibia without spinous processes and tarsus 2 composed of 9 articles	*Newportia heteropoda*
–	Femur of ultimate legs with 4 spinous processes, tibia with 2 spinous processes (Figs 9, 10) tarsus 2 of 19 articles (Fig. 8)	*Newportia stoevi* sp. n.
18	Tergite 1 with complete paramedian sutures which cross the anterior transverse suture	*Newportia longitarsis longitarsis*
–	Tergite 1 with incomplete paramedian sutures of various length, from half-complete (Fig. 17) to rudimentary (Fig. 13), running between the posterior tergal margin and the anterior transverse suture; rarely without any trace of paramedian sutures	19
19	Cephalic plate with a thin transverse suture which crosses the short paramedian sutures close to the posterior margin of the cephalic plate	*Newportia longitarsis sylvae*
–	Cephalic plate without any transverse suture	20
20	Prefemur of ultimate legs with 3 large ventral spinous processes	*Newportia longitarsis guadeloupensis*
–	Prefemur of ultimate legs with 4 large ventral spinous processes	*Newportia longitarsis stechowi*
21	Tarsus 2 of ultimate legs composed of 39–40 articles	*Newportia sabina*
–	Tarsus 2 of ultimate legs composed of less than 30 articles	22
22	Each leg with a tarsal spur; tibia of ultimate legs longer than femur	23
–	Legs without tarsal spurs; tibia of ultimate legs shorter or as long as femur	26
23	Femur of ultimate legs with 2 ventral spinous processes	*Newportia morela*
–	Femur of ultimate legs with 3 ventral spinous processes	24
24	Outer branches of forked paramedian sutures of tergite 1 extending in front of the anterior transverse suture up to the anterior border of this tergite	*Newportia spinipes* + *Newportia azteca*
–	Forked paramedian sutures of tergite 1 ending up in the anterior transverse suture	25
25	Tarsus 2 of ultimate legs consists of 11–12 articles, prefemur laterally with strong setae	*Newportia atoyaca*
–	Tarsus 2 of ultimate legs consists of 5–8 articles, prefemur laterally with small spines	*Newportia oriena*
26	Tarsus of ultimate legs composed of uniform articles (Fig. 18)	27
–	Tarsus 1 and tarsus 2 of ultimate legs with different shapes (Fig. 19)	29
27	Tergite 1 with paramedian sutures in front of the anterior transverse suture (Fig. 14); tibia of ultimate legs cylindrical, tarsus composed of 7–9 articles (Fig. 18)	*Newportia ignorata*
–	Tergite 1 without paramedian sutures in front of the anterior transverse suture; tibia of ultimate legs distinctly claviform distally (Fig. 20), tarsus composed of ca. 15 articles (*Newportia weyrauchi*)	28
28	Prefemur of ultimate legs with 4 ventral spinous processes	*Newportia weyrauchi weyrauchi*
–	Prefemur of ultimate legs with 3 ventral spinous processes	*Newportia weyrauchi thibaudi*
29	Outer branches of the forked paramedian sutures of tergite 1 crossing the anterior transverse suture (Fig. 14) and ending onto the tergal anterior margin	30
–	Forked paramedian sutures of tergite 1 ending in the anterior transverse suture	*Newportia monticola*
30	Femur of ultimate legs medially with one basal and one distal spinous processes, ventrally without them. Sternites 2-12(15) each with a median longitudinal sulcus	*Newportia fuhrmanni*
–	Femur of ultimate legs medially with one basal spinous process, ventrally with 1-2 such processes. Sternites 2-19 each with a median longitudinal sulcus	*Newportia simoni*
31	Ultimate legs with a well-developed claw-shaped pretarsus (which is as long as the poorly distinct ultimate article of tarsus 2)	*Newportia amazonica*
–	Ultimate legs normally without claw-shaped pretarsus (if a small ultimate claw is present, then it is less than half as long as the poorly distinct ultimate article of tarsus 2)	32
32	Tibia of ultimate legs with 3 ventral spinous processes; medial spinous processes of prefemur almost as large as the ventral ones	*Newportia mexicana*
–	Tibia of ultimate legs without spinous processes, medial spinous processes of prefemur (when present) considerably smaller than the large ventral ones	33
33	Tergite 1 with rounded anterior transverse suture and with or without paramedian sutures	34
–	Tergite 1 with anterior transverse suture angulated caudad to midline and giving rise to short longitudinal suture, which bifurcate caudally (fig. 1 in Chagas and Shelley 2003)	*Newportia troglobia*
34	Tergite 1 without paramedian sutures between its posterior margin and the anterior transverse suture, rarely with very short tracks just behind the anterior transverse suture (Fig. 13)	35
–	Tergite 1 with complete (more rarely half-complete) paramedian sutures	37
35	Paramedian sutures of cephalic plate very short. Forcipular coxosternite without median suture. Ultimate sternite without median longitudinal sulcus. Femur of ultimate legs with a row of 2-3 spinous processes	36
–	Paramedian sutures of cephalic plate almost reaching its middle. Forcipular coxosternite with a well-developed median suture. Ultimate sternite with a clear median longitudinal sulcus or depression. Femur of ultimate legs without or with a single very small spinous process	*Newportia maxima*
36	Tarsus of ultimate legs uniform, without distinct division into tarsus 1 and 2. Forcipular trochanteroprefemur without process	*Newportia lasia*
–	Tarsus of ultimate legs distinctly divided into a shorter tarsus 1 and a longer tarsus 2 (Fig. 21). Forcipular trochanteroprefemur with a process	*Newportia patavina*
37	Paramedian sutures of tergite 1 not bifurcate	38
–	Paramedian sutures of tergite 1 bifurcate	*Newportia pelaezi*
38	Anterior transverse suture of tergite 1 interrupted between the paramedian sutures (Fig. 22)	*Newportia divergens*
–	Tergite 1 with a complete anterior transverse suture (Figs 15, 17)	39
39	Tarsus of ultimate legs uniform	*Newportia brevipes*
–	Tarsus 1 of ultimate legs abruptly differing from the tarsus 2	40
40	Cephalic plate with a transverse suture crossing the paramedian sutures near the posterior margin (*Newportia ernsti*)	41
–	Cephalic plate without transverse suture (Fig. 13)	42
41	Prefemur of ultimate legs with 6 (rarely 7) ventral spinous processes. Transverse suture of cephalic plate very distinct	*Newportia ernsti ernsti*
–	Prefemur of ultimate legs with 5 ventral spinous processes. Cephalic plate: median part of transverse suture between the paramedian sutures often poorly visible	*Newportia ernsti fossulata*
42	Paramedian sutures of tergite 1 ending up in the anterior transverse suture; tergite 2 with complete paramedian sutures	*Newportia bielawskii*
–	Paramedian sutures of tergite 1 complete, crossing the anterior transverse suture; tergite 2 with short paramedian sutures or sutures totally missing	43
43	Tergite 2 without paramedian sutures (these begin from tergite 5-6 onwards); four basal articles of tarsus 2 of ultimate legs definitely separated from each other (fig. 9 in Chamberlin 1957)	*Newportia albana*
–	Tergite 2 with shortened paramedian sutures (Fig. 22); all articles of tarsus 2 of ultimate legs not well separated	44
44	All legs with a tarsal spur and both lateral and ventral tibial spurs	*Newportia stolli*
–	All legs with a lateral tibial spur only	*Newportia paraensis*

**Figures 13–16.**
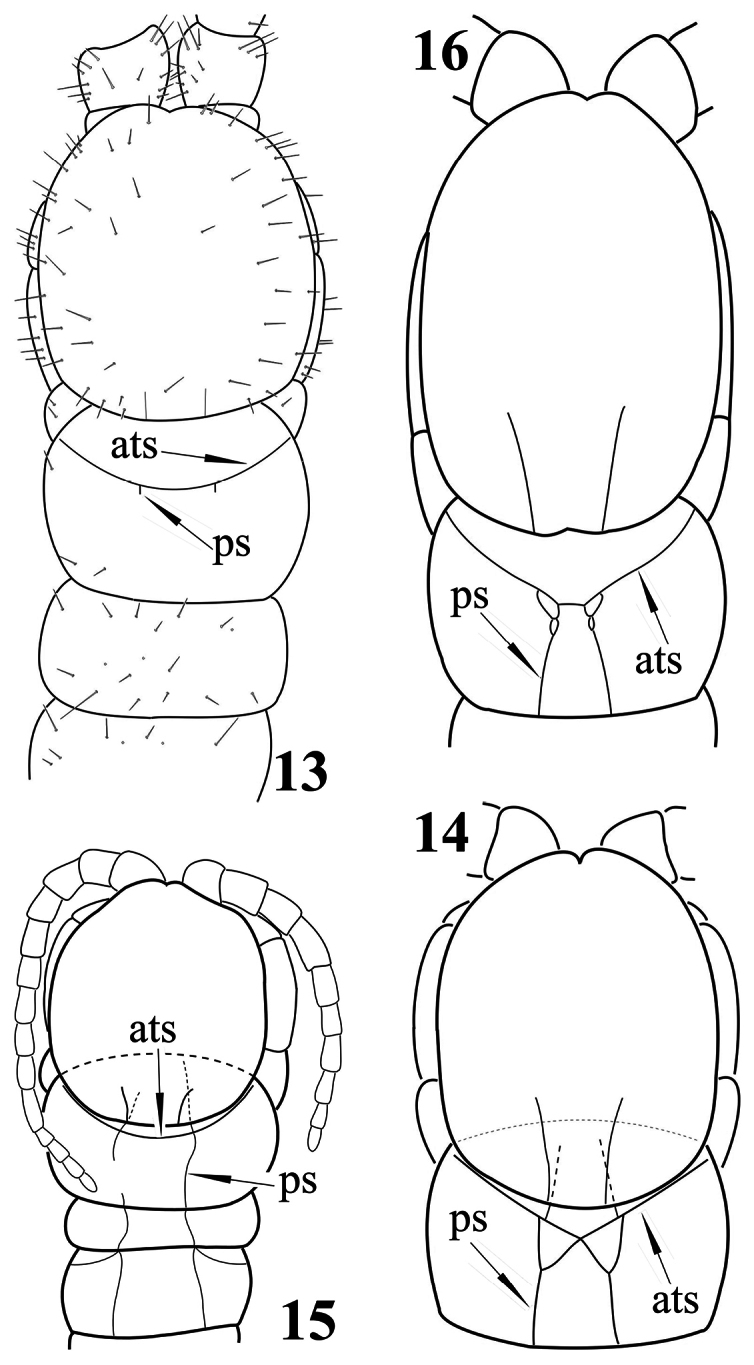
*Newportia* sp. **13** Cephalic plate and tergites 1-3, dorsal view (after Schileyko and Minelli 1998, re-drawn); *Newportia ignorata* Kraus, 1955 **14** Cephalic plate and tergite 1, dorsal view (after Schileyko and Minelli 1998); *Newportia diagramma* Chamberlin, 1921 **15** Cephalic plate and tergites 1-3, dorsal view (after Schileyko and Minelli 1998); *Newportia oligopla* Chamberlin, 1945 **16** Cephalic plate and tergite 1, dorsal view (after Chamberlin 1945, re-drawn); (**ats**) – anterior transverse suture, (**ps**) – paramedian suture.

**Figures 17–20.**
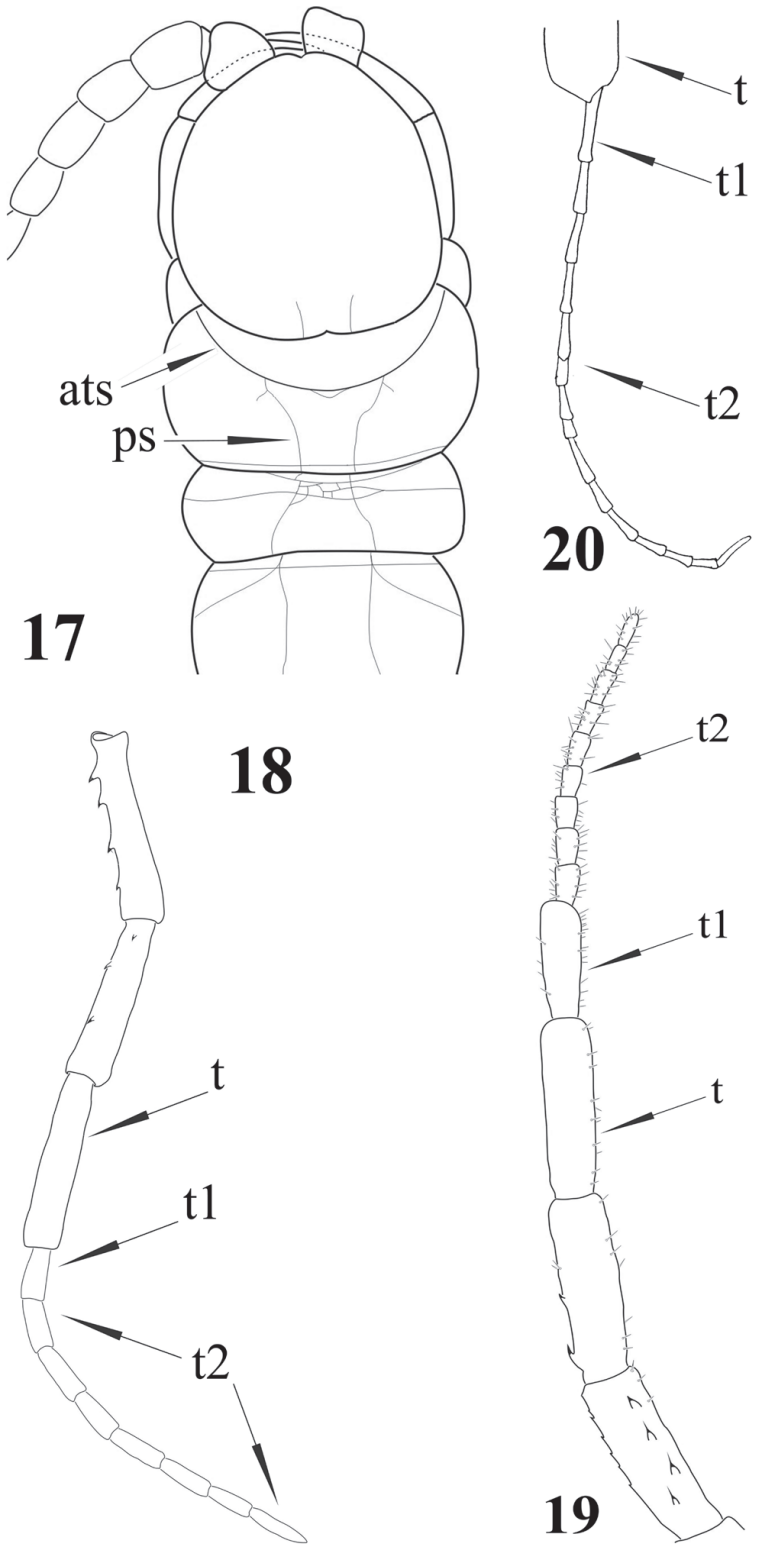
*Newportia adisi* Schileyko & Minelli, 1998 **17** Cephalic plate and tergites 1-3, dorsal view (after Schileyko and Minelli 1998); *Newportia ignorata* Kraus, 1955 **18** Right ultimate leg, medially (after Schileyko and Minelli 1998); *Newportia monticola* Pocock, 1890 **19** Right ultimate leg, ventral view (after Schileyko and Minelli 1998); *Newportia weyrauchi* Chamberlin, 1955 **20** Ultimate leg: distal portion of tibia and tarsus, ventral view (after Chamberlin 1955); (**ats**) – anterior transverse suture, (**ps**) – paramedian suture, (**t**) – tibia, (**t1**) – tarsus 1, (**t2**) – tarsus 2.

**Figures 21, 22.**
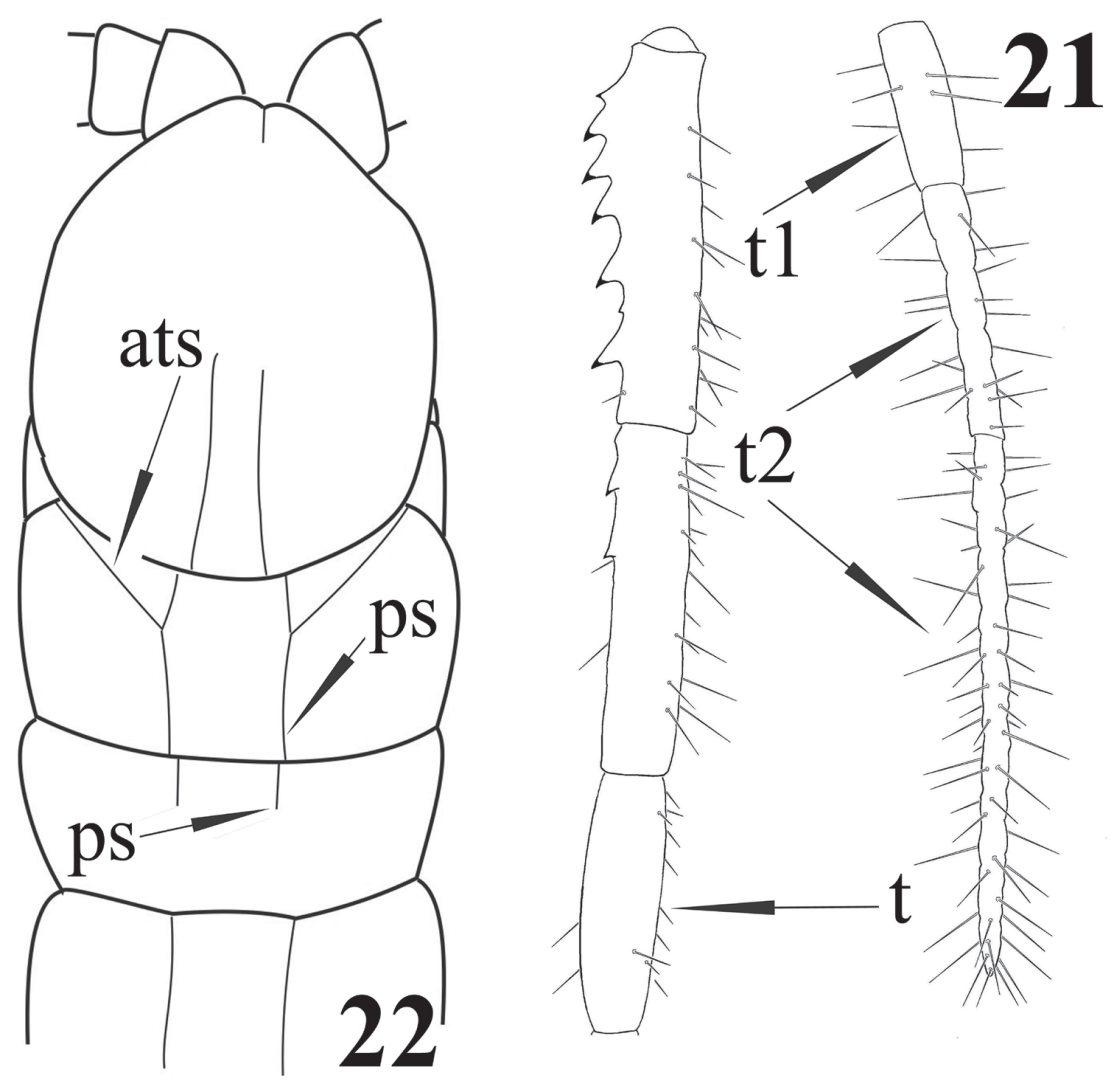
*Newportia patavina* Schileyko & Minelli, 1998 **21** Right ultimate leg, medially (after Schileyko and Minelli 1998); *Newportia divergens* Chamberlin, 1922 **22** Cephalic plate and tergites 1-3, dorsal view (after Chamberlin 1922, re-drawn); (**ats**) – anterior transverse suture, (**ps**) – paramedian suture, (**t**) – tibia, (**t1**) – tarsus 1, (**t2**) – tarsus 2.

## Supplementary Material

XML Treatment for Newportia stoevi
